# A Case of Peritoneal Dialysis-Related Peritonitis Caused by *Ewingella americana*

**DOI:** 10.1155/2022/5607080

**Published:** 2022-02-18

**Authors:** Catarina Abrantes, Joana Freitas, Tânia Silva, Luís Marques da Silva, Maria João Carvalho, Anabela Rodrigues, António Cabrita

**Affiliations:** ^1^Nephrology Department, Setubal Hospital Center, Setubal, Portugal; ^2^Nephrology Department, Hospital de Santo António, University Hospital Center of Porto, Porto, Portugal; ^3^Microbiology Department, Hospital de Santo António, University Hospital Center of Porto, Porto, Portugal

## Abstract

Peritoneal dialysis (PD)-related peritonitis is a frequent complication. PD units should be aware of all possible pathogens and share their experience about prevention and optimal management. Uncommon bacteria, a special group with crescent incidence in PD practice, may require singular considerations. A case of peritonitis due to *Ewingella americana,* a rare human pathogen, is reported, with a favorable outcome. To date, only three other cases have been described in the literature. New evidence is necessary for a better understanding of this pathogen and its consequences in PD modality.

## 1. Introduction

Peritonitis is a frequent and potential serious complication of peritoneal dialysis (PD), directly related to adverse outcomes, including technique failure and mortality [1, 2]. Prevention of PD-associated peritonitis should, therefore, be a focus of every PD unit. Knowing the source of peritonitis, including transmission patterns of pathogens, is essential for a personalized approach when it comes to retraining the patient after an infection. For example, coagulase-negative staphylococcal species and *Staphylococcus aureus*, known colonizers of human skin, are responsible for the majority of PD-related peritonitis cases [3, 4]. Atypical organisms, on the contrary, show increasing relevance in our daily practice and must be dealt with care, particularly in patients from impoverished or rural environments, where habitational context takes a special importance.

## 2. Case Description

A 45-year-old female patient on PD for the past 2 years was admitted to the hospital with diffuse abdominal pain and vomiting. She lived in a rural area, and her background was relevant for end-stage renal disease due to IgA nephropathy. Presently, she was on automated PD (APD), and her previous PD history included one peritonitis episode due to *Streptococcus salivarius,* with a favorable outcome after a course of intravenous vancomycin and one chronic exit-site infection requiring removal of the PD catheter and temporary transition to hemodialysis before a new catheter could be safely inserted.

On admission, she was afebrile (tympanic temperature 36.4°C), with stable vital signs (blood pressure 127/77 mmHg and pulse 83/min). Physical examination revealed abdominal tenderness without rebound discomfort, and peritoneal dialysate was hazy at macroscopic observation. Inspection of the exit site did not show signs of infection. Laboratory workup showed a white blood cell (WBC) count of 14.1 × 10^9^/L (12.3 × 10^9^/L neutrophils) and C-reactive protein (CRP) of 225.12 mg/L, and peritoneal dialysate analysis revealed a WBC count of 7261 cells/*μ*L with polymorphonuclear predominance (6252 cells/L). A diagnosis of peritonitis was established, and empirical treatment with intravenous vancomycin and intraperitoneal (IP) ceftazidime was started (Table 1).

Samples of dialysate were obtained and sent to the microbiology department for analysis. After 48 hours of incubation, Gram-negative bacilli were detected on Gram staining. Identification of the bacteria was initially performed using the conventional VITEK 2^™^ automated system and later confirmed by MALDI-TOF MS having both identified the bacilli as *Ewingella americana*. Blood cultures were negative. Antimicrobial susceptibility was performed and found ampicillin, ceftazidime, trimethoprim/sulfamethoxazole, and ciprofloxacin to be effective against this microorganism. After these results, vancomycin was discontinued, and the patient completed a 3-week course of IP ceftazidime, with total recovery.

When questioned about the PD technique, the patient denied shortcuts or inadequate hygiene, but referred using water from a nearby fountain as domestic water.

## 3. Discussion

*Ewingella americana* was first described in 1983 by Grimont et al. and its generic name honors American bacteriologist William Ewing, while the species name refers to the American source of the clinical isolates described. It is a rare member of the order Enterobacterales and the only known species in the genus. The rarity of reported infections in humans raised initial doubts as to its true pathogenicity. However, though sparse and scattered in time, increasing reports have confirmed clinical infections due to *E. americana* in multiple contexts, including bacteremia [6–9], pneumonia [10], conjunctivitis [11, 12], Waterhouse–Friderichsen syndrome [13], and peritonitis [14–16]. Susceptible populations include immunocompromised patients, but previously healthy patients were described too.

A recent review by Khurana et al. [16] showed only three reported cases of peritonitis secondary to *E. americana*. To the best of our knowledge, this is the fourth worldwide peritonitis caused by this organism and the first ever reported in Portugal. According to Khurana et al., all three previous cases occurred in female patients, as in our case; however, ours is much younger comparatively. The previous cases were found to be nonsusceptible to commonly used empiric antibiotics, such as cephalosporins, but not in our case, although all patients had a favorable outcome, without the need of catheter removal.

Despite not being a recently discovered organism, little is known about its natural habitat. Available data from case reports suggest that *E. americana* survives without relevant nutritional needs and preferably grows at 4°C. It was also proposed in two case reports that water could be a reservoir for this pathogen [14, 16]. Similarly, in our case, despite the fact that the source of this Gram-negative microorganism remains undetermined, we may presume that use of contaminated water and break in sterile technique could help explaining this infection. As stated before, prevention of PD-associated peritonitis is a key feature in patient management, and proper care of the catheter exit site plays a pivotal role in prevention. The patient previous PD history was suggestive of a precarious technique, which corroborates this assumption.

## 4. Conclusion

We describe a rare case of peritonitis due to *Ewingella americana* in a patient on CAPD, the first ever reported in Portugal and the fourth worldwide. To date, there is limited evidence concerning the natural habitat of this organism and its clinical significance in humans. Still, available reports account for a nonaggressive and treatable infection, with a favorable outcome. Future studies are needed to clarify the clinical potential of *E. americana* and its ecology, including possible role of contaminated water as the source of this pathogen.

## Figures and Tables

**Table 1:** Biochemical data on admission and at 72 hours.

	On admission	Day 3	Reference values
Hemoglobin (g/dL)	11.5	11.3	13–17
Hematocrit (%)	38	37	40–50
Platelet (10^3^/*μ*L)	256	312	150–350
Plasma WCC (10^9^/*μ*L)	14.1	10.6	4.5–11.4
Neutrophils (10^9^/*μ*L)	12.3	7.2	
Sodium (mEq/L)	139	140	136–146
Potassium (mEq/L)	4.7	4.6	3.5–5.1
Chloride (mEq/L)	103	103	98–107
Phosphorus (mg/dL)	4.2	—	2.7–4.5
Serum calcium^*∗*^ (mg/dL)	9.14	—	8.4–10.2
Magnesium (mg/dL)	2.1	—	1.6–2.6
Glucose (mg/dL)	114	—	70–105
GOT (U/L)	31	—	<38
GPT (U/L)	40	—	<41
Albumin (g/dL)	3.3	—	3.5–5.0
CRP (mg/L)	225.12	57.10	<0.5
WCC in PD fluid (cell/*μ*L)	7261	287	<100
PMN in PD fluid (cell/*μ*L)	86.1	31.8	<50%

^*∗*^Corrected for albumin calcium levels. CRP: C-reactive protein; GOT: glutamic oxaloacetic transaminase; GPT: glutamic pyruvic transaminase; PMN: polymorphonuclear; WCC: white cell count.

## Data Availability

The literature used to support the findings of this case report is included within the article.
